# Postnatal development and maturation of layer 1 in the lateral prefrontal cortex and its disruption in autism

**DOI:** 10.1186/s40478-019-0684-8

**Published:** 2019-03-13

**Authors:** Iris Margalit Trutzer, Miguel Ángel García-Cabezas, Basilis Zikopoulos

**Affiliations:** 1 Human Systems Neuroscience Laboratory, Boston University, 635 Commonwealth Ave., Room 401D, Boston, MA 02215 USA; 2 Program in Neuroscience, Boston University, Boston, MA 02215 USA; 3 Neural Systems Laboratory, Boston University, Boston, MA 02215 USA

**Keywords:** Autism neuropathology, Laminar architecture, Postnatal axon myelination, Feedback pathways, Inhibitory neuron, Anterior cingulate cortex

## Abstract

Autism is a neurodevelopmental connectivity disorder characterized by cortical network disorganization and imbalance in excitation/inhibition. However, little is known about the development of autism pathology and the disruption of laminar-specific excitatory and inhibitory cortical circuits. To begin to address these issues, we examined layer 1 of the lateral prefrontal cortex (LPFC), an area with prolonged development and maturation that is affected in autism. We focused on layer 1 because it contains a distinctive, diverse population of interneurons and glia, receives input from feedback and neuromodulatory pathways, and plays a critical role in the development, maturation, and function of the cortex. We used unbiased quantitative methods at high resolution to study the morphology, neurochemistry, distribution, and density of neurons and myelinated axons in post-mortem brain tissue from children and adults with and without autism. We cross-validated our findings through comparisons with neighboring anterior cingulate cortices and optimally-fixed non-human primate tissue. In neurotypical controls we found an increase in the density of myelinated axons from childhood to adulthood. Neuron density overall declined with age, paralleled by decreased density of inhibitory interneurons labeled by calretinin (CR), calbindin (CB), and parvalbumin (PV). Importantly, we found PV neurons in layer 1 of typically developing children, previously detected only perinatally. In autism there was disorganization of cortical networks within layer 1: children with autism had increased variability in the trajectories and thickness of myelinated axons in layer 1, while adults with autism had a reduction in the relative proportion of thin axons. Neurotypical postnatal changes in layer 1 of LPFC likely underlie refinement of cortical activity during maturation of cortical networks involved in cognition. Our findings suggest that disruption of the maturation of feedback pathways, rather than interneurons in layer 1, has a key role in the development of imbalance between excitation and inhibition in autism.

## Introduction

Autism is a neurodevelopmental disorder that is characterized by changes in neural communication that affect diverse sensory-motor processes such as attention and social interaction [2, 36, 66]. Changes in frontal networks, including increased short-range and decreased long-range communication as well as changes in synchronization between cortical areas during tasks, have been described in individuals with autism [12, 24, 31, 61, 63, 110, 124]. Anatomical studies have identified changes in the distribution and density of neurons belonging to multiple subtypes within frontal cortices [1, 52, 130] and myelinated axons below the frontal lobes in autism [129, 130, 133] that likely underlie these findings. However, little is known about the development of cortical pathology and the disruption of laminar-specific excitatory pathways and inhibitory circuits in the affected frontal cortical networks.

The development of cortical network pathology in the lateral prefrontal cortex (LPFC) is of particular interest because LPFC is involved in attention and the cognitive processes that are affected in autism and undergoes prolonged postnatal development and maturation [13, 23, 52, 71, 115, 116, 129–131]. Layer 1 plays a significant role in the prenatal patterning of the cortex and postnatally is a chief recipient of feedback and neuromodulatory pathways in LPFC, making it an ideal candidate for the study of the development of laminar-specific pathway pathology in autism.

Layer 1 contains a distinctive set of morphologically diverse local circuit neurons along with varied populations of astrocytes, oligodendrocytes, and microglia [11, 40, 81, 97, 102, 125, 127]. Feedback connections from cortical areas as well as the thalamus, amygdala, and neuromodulatory systems target layer 1 [5, 7, 10, 15, 48, 60, 87, 112, 128], where they interact with local excitatory and inhibitory circuits and affect spatiotemporal characteristics of cortical activity patterns [17, 26, 33]. In prenatal development, the intrinsic Cajal-Retzius cells of layer 1 secrete reelin to direct the development of the distinct cortical layers [39, 53, 93]. Studies of the development of layer 1 have examined mostly the pre- and postnatal maturation of Cajal-Retzius neurons and few other cell types [78, 82, 88, 90, 102, 114, 126, 127]. However, we know little about the postnatal changes in the diverse cellular populations of layer 1 and their relationship with the maturation of the pathways that terminate there, which serve to transition this layer from a developmental mediator to a processor of feedback input. Importantly, changes in the expression of factors that determine the maturation and activity of cortical networks have been described in layer 1 neurons in LPFC in neurodevelopmental connectivity disorders such as schizophrenia [104] and autism [115]. The effects of these disruptions on the cellular organization and axonal networks within layer 1 in childhood and adulthood are unknown.

To begin to address these issues we used post-mortem brain tissue from individuals with and without autism (ages 3–67 years) to systematically and quantitatively examine the postnatal development of excitatory and inhibitory circuits in layer 1 of the human LPFC. We cross-validated our findings through comparisons with neighboring anterior cingulate cortices (ACC) of neurotypical individuals and optimally-fixed non-human primate tissue. We provide evidence for significant changes in the density of neurons and maturation of pathways in typical postnatal development from childhood through late adulthood. We notably found that PV-immunoreactive neurons, which have previously only been shown in layer 1 of neonates, persist in layer 1 through late adolescence, likely influencing the signaling dynamics of the inhibitory neurons in layer 1. Comparison of the structure of layer 1 in typically developing individuals with that of individuals with autism revealed significant disruption in the developmental trajectory and structure of pathways, but not neurons, in children and adults with autism. Pathology included increased variability in axon orientation and changes in the size of myelinated axons. These findings suggest that persistent disruption of feedback pathways in LPFC in children and adults may underlie excitatory-inhibitory imbalance, cortical network disorganization, and atypical focusing and shifting of attention in autism.

## Methods

### Experimental design

The aim of this study was to examine the structural, neurochemical, and molecular characteristics of layer 1 of the human LPFC in children and adults with and without a diagnosis of autism, a disorder in which imbalance of excitation-inhibition has been shown using multiple approaches [27, 49, 103]. First, we examined the postnatal structural and neurochemical maturation of layer 1 in neurotypically developing individuals to define the typical developmental trajectory. We compared typical development with the trends seen in children and adults with autism in order to probe whether there were patterns of cortical disorganization that could underlie autism symptomatology.

**Fig. 1:**
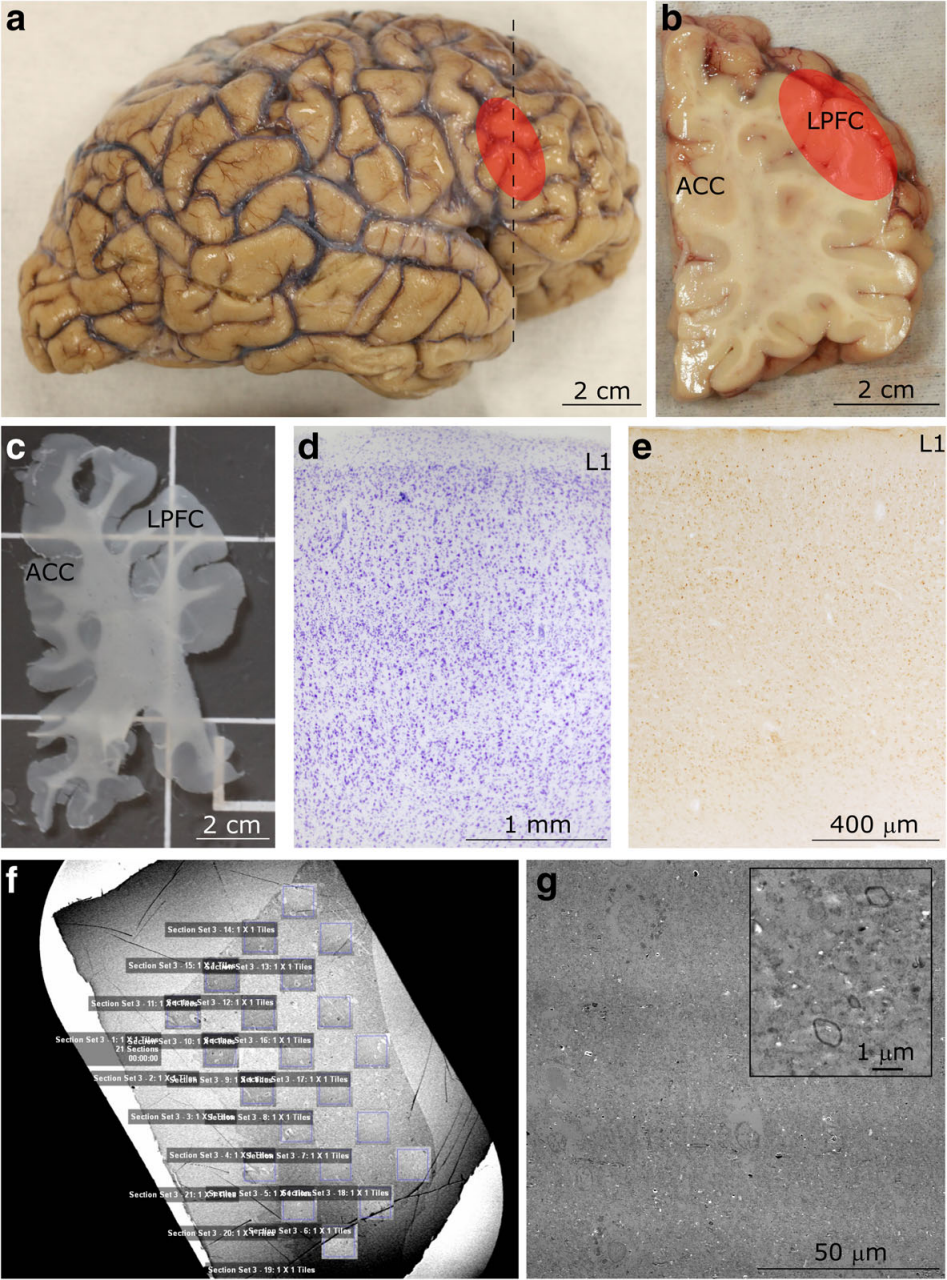
Experimental design. **a** Lateral view of the right hemisphere of the adult human brain. The region of the lateral prefrontal cortex (LPFC) analyzed in this study is shown in red. **b** Coronal tissue slab taken from the frontal cortex at the level marked by the dotted line in **a**. The red overlay highlights the LPFC at the level of Brodmann’s area 46. **c** Representative free-floating tissue section, cut at 50 μm, used for staining. **d-e** Representative cortical columnar regions of interest processed for Nissl **d** and calretinin **e** in the LPFC. Layer 1 (L1) is labeled in both columns. **f** Representative image of a 50 nm-thick section from LPFC gray matter on a pioloform-coated slot grid to show the unbiased systematic sampling scheme used to analyze axons at the EM level. This scheme also resembles the sampling used in quantitative analysis of cell and axon populations at the light microscope. **g** Representative high-resolution electron micrograph of an ultrathin section (50 nm), sampled from **f**, and acquired using a scanning-transmission electron microscope (STEM) system. Myelinated axon profiles can be identified by the darkly stained, electron dense ring of myelin surrounding the axolemma (shown at high magnification in inset). This representative image was taken from layer 5 of LPFC

We focused our analysis specifically on layer 1 of LPFC because it is a major target of feedback pathways and is involved in the performance of high-order organizational tasks and attentional regulation, which are affected in autism. We systematically and quantitatively examined neurons and myelinated axons in layer 1 of LPFC and separately analyzed the excitatory and inhibitory components of the local neural network (Fig. 1). To describe the typical range of fundamental anatomical and neurochemical features of layer 1, we qualitatively studied and compared layer 1 of neurotypical subjects in the LPFC and in the ACC, two regions of the prefrontal cortex with different laminar structures [131]. We used archival processed tissue from ACC and LPFC of rhesus macaques as an important control to validate the results from the analysis of human tissue, and placed our findings within the context of classical and modern studies of the cellular composition, circuitry, and connectivity of layer 1. Tissue was labeled with Nissl and Gallyas stain to identify the overall structural characteristics of layer 1, and we used specific antibodies to label glia, inhibitory neurons, and markers of cortical stability and plasticity. Within this framework of analyses we were able to describe dynamic changes in network structure during neurotypical development, and we were able to identify pathological changes in patterns of cortical organization underlying information processing during development in autism.

### Post-mortem tissue acquisition and processing

Post-mortem age-matched tissue from 32 individuals (16 control, 16 autism) was acquired from the Harvard Brain Tissue Resource Center, the Autism Tissue Program, the Institute for Basic Research in Developmental Disabilities, the University of Maryland Brain and Tissue Bank, and the National Disease Research Interchange (NDRI). Autism diagnosis was assessed through the Autism Diagnostic Interview-Revised (ADI-R) and many cases had corresponding Autism Diagnostic Observation Schedule (ADOS) scores. Four subjects had comorbid diagnoses of seizure disorder (HSB4640, AN 08792), depression (AN 18892) and schizophrenia (AN 06746); findings in these cases, which have also been successfully used in previous studies [133], fell within the ranges for each group. All available cases were included in the analyses for this study. Case AN18892 (B-4871) was used solely in a qualitative assessment of ACC. All other cases were included in quantitative immunohistochemistry analyses. Cases 4021, 4029, AN 03221, 5144, 1182, B-5173, B-6232, B-6677, 451, 4203, 4337, 3835, B-6004, B-5353, B-4981, HAW, and HAY were used for electron microscopy analyses. Clinical characteristics and other data on human subjects can be found in Table 1.

**Table 1:** Cases and Clinical Characteristics

Subject Number	Age (years)	Condition	Sex	PMI (h)	Cause of Death
AN03345 (B-6399)	3	AU	M	4	Cardiac arrest
4021	3.3	AU	M	15	Accidental drowning
4029	3.8	AU	M	13	Accidental drowning
5308	4.5	AU	M	21	Accident, injuries
AN08873 (B-5569)	5	AU	M	25.5	Accidental drowning
AN03221	7	AU	M	11	Accidental drowning
5144	7.2	AU	M	3	Cancer
HSB4640	8.5	AU	M	14	Asthma attack, seizure
AN01293 (B-6349)	9	AU	M	4	Cardiopulmonary arrest
1182	10	AU	F	24	Smoke inhalation
AN04682 (B-7079)	15	AU	M	23.2	Asphyxia, hanging
AN-08792 (B-5173)	30	AU	M	20	Gastro-intestinal bleeding
AN-11989 (B-6677)	30	AU	M	16	Congestive heart failure
AN-18892 (B-4871)	31	AU	M	99	Shooting
AN-07770 (B-6232)	40	AU	F	33	Respiratory arrest
AN-06746 (B-4541)	44	AU	M	31	Acute myocardial infarction
451	4.6	CTR	M	15	Accidental drowning
4203	7.8	CTR	M	24	Respiratory insufficiency
4337	8.2	CTR	M	16	Blunt force neck injury
1706	8.6	CTR	F	20	Rejection of cardiac transplant
3835	9.6	CTR	F	8	Asphyxia
1548	10	CTR	M	0	Unknown
1670	14	CTR	M	5	Respiratory distress
4722	14	CTR	M	16	Multiple traumatic injuries
B-4786	36	CTR	M	20	Myocardial infarction
B-6004	36	CTR	F	18	Unknown
HCD	38	CTR	M	18.9	Cardiac arrest
B-5353	41	CTR	F	14	Unknown
B-4981	42	CTR	M	18	Myocardial infarction
HCF	47	CTR	M	21.9	Cardiac arrest
HAW	58	CTR	F	30	Pancreatic cancer
HAY	67	CTR	M	30	Pancreatic cancer

PMI: Post-mortem Interval

Cases were matched based on availability, and brains were well preserved and had low post-mortem interval (PMI) (average PMI < 24 h). To ensure appropriate age-matching, HAW and HAY, which are older control cases, were excluded from the comparison with individuals with autism but were included in the analysis of neurotypical development and aging [average age of individuals with autism: Children = 6.9 ± 3.6 years (1 F), Adults = 36.0 ± 7.1 years (1 F); average age of age-matched neurotypical individuals: Children = 9.5 ± 3.2 years (2 F), Adults = 40.0 ± 4.2 years (2 F)]. Tissue was fixed in 10% formalin, cryoprotected in 30% sucrose, and stored at -20^o^ C in antifreeze solution (30% ethylene glycol, 30% glycerol, 40% 0.05 M PB with 0.05% azide).

We evaluated layer 1 in granular LPFC and dysgranular ACC in order to identify the fundamental composition of layer 1, which we assessed by noting both common organizational patterns and distinct features of areas that otherwise have gross differences in laminar organization. In adult cases, coronal blocks of tissue corresponding to area 46 in the LPFC or area 32 in the ACC were identified using atlases as guides and were excised for sectioning [6, 77, 100, 101, 122, 123]. In children, coronally-cut slabs of the frontal lobe were divided into dorsal and ventral blocks and sectioned for analysis. Sections from equivalent levels from all cases were matched to control for structural differences along the rostro-caudal axis of our region of interest (ROI). Tissue blocks were frozen in -70^o^ C isopentane to prepare for sectioning into 10 series of 50 μm-thick sections using a cryostat (CM 1500, Leica) or freezing microtome (American Optical). Few sections from cases B-6232 and B-6677 were also cut at 20 μm and stained for Nissl analysis. Results from these thin sections did not differ from those obtained from thicker sections. Alternatively, additional blocks from adult cases that were not frozen were rinsed in 0.1 M PB and cut in series of 50 μm-thick sections using vibratomes (series 1000, Pelco; Precisionary VF-700, Precisionary Instruments Inc.). One series of sections was mounted immediately following sectioning on chrome-alum gelatin-coated slides in order to preserve the series. These sections were used as a reference to determine the position of floating sections on the rostro-caudal axis.

We evaluated layer 1 in LPFC and ACC in optimally-fixed brain tissue from non-human primates (*Macaca mulatta*) as an important control in order to assess the effects of PMI and quality of fixation on our quantitative analysis and to further cross-validate our findings. We obtained archival tissue from young adult rhesus monkeys (aged 2–4 years) that were used for experiments and cared for in accordance with the requirements of the Institutional Animal Care and Use Committee (IACUC) of Boston University. Monkey cases RAJ (F), RAN, RAT (2 years old, F), RAV, RAW (F), RAX, RAY (3 years old, F), RBB (2 years old, F), RBI (2.8 years old, F), RBN (2 years old, M), RBO (3 years old, M), RBS (3.5 years old, F), RBT (4 years old, F) and RBU (4 years old, M) were used for analyses. Monkeys were perfused with 4% paraformaldehyde (RAJ, RAN, RAT, RAV, RAW, RBB) or 4% paraformaldehyde with 0.2% glutaraldehyde (RAX, RAY, RBI, RBN, RBO, RBS, RBT, RBU) as previously described [46, 131]. The brains of these animals were then cryoprotected in graded sucrose solutions and frozen in -70 °C isopentane for rapid, uniform freezing. Brains were sectioned into 40 μm-thick sections (RAJ, RAN, RAT, RAV, RAW, RAX, RAY) or 50 μm-thick sections (RBB, RBI, RBN, RBO, RBS, RBT, RBU) in 10 matched series, with one series mounted immediately for reference, as described above [46].

### Histology and immunohistochemistry for light microscopy

Nissl-stained sections were used to analyze overall neuron density in layer 1. Sections were stained as previously described [46]. Briefly, sections underwent de-fatting in a 1:1 solution of chloroform and 100% ethanol for 2–3 h prior to rehydration using a gradient of alcohols and dH_2_O. Sections were stained using 0.05% thionin blue for 15 min. Stained tissue was dehydrated and differentiated in graded alcohols and xylenes prior to coverslipping using Entellan (Merck).

Gallyas myelin labeling of human tissue followed published protocols [43, 132]. Briefly, sections were rinsed in distilled water and then incubated in pyridine (2/3; P368–1 Fischer Scientific) with glacial acetic acid (1/3; ARK2183 Sigma-Aldrich) for 30 min at room temperature. Sections were washed again in distilled water followed by incubation in the impregnation solution [0.1 g ammonium nitrate (A7455 Sigma-Aldrich) and 0.1 g silver nitrate (S181–25 Fischer Scientific) per 100 ml of distilled water, pH 7.5] for a minimum of 30 min at room temperature in the dark. Sections were rinsed in 0.5% acetic acid (A6283 Sigma-Aldrich) and were then incubated in the developing solution [Three components: A, 25 g sodium carbonate (S-263 Fischer Scientific) in 500 ml distilled water; B, 1 g ammonium nitrate (A7455 Sigma-Aldrich), 1 g silver nitrate (S181–25 Fischer Scientific) and 5 g silico-tungstic acid (383,341 Sigma-Aldrich) in 500 ml distilled water; C, 75 ml of solution B and 1.75 ml of 4% paraformaldehyde (O4042 Fischer Scientific) in 500 ml distilled water] under microscopic control until the proper level of stain was achieved. 150 ml of solution A was combined with 75 ml of solution B and 75 ml of solution C, in that order, to create the impregnation solution. After developing, sections were washed in 1% acetic acid (A6283 Sigma-Aldrich) and then in distilled water before incubation in 5% sodium thiosulfate (S-1648 Sigma-Aldrich) to stabilize the reaction. Sections were finally washed in distilled water and mounted and coverslipped as described above.

Inhibitory interneurons and glial cells in human tissue were labeled through immunohistochemistry as previously reported [32, 47, 84, 130]. Three functionally distinct classes of interneurons are distinguished in the primate cortex through their differential expression of calcium-binding proteins. Calbindin (CB)-expressing neurons target the distal dendrites of pyramidal neurons, while calretinin (CR)-expressing interneurons target CB neurons [28]. Parvalbumin (PV)-expressing interneurons synapse on the perisomal regions of their targets and are therefore strongly inhibitory [28]. We labeled glial cells with antibodies against CD44 (to label interlaminar astrocytes), excitatory amino acid transporter-2 (EAAT2, to label astrocytes), and ionized calcium-binding adaptor molecule 1 (Iba-1, to label microglia). Information regarding the antibodies used in this study is included in Table 2.

**Table 2:** Properties of the Antibodies Used

Antigen	Animal Raised	Source	Catalog Number	Research Resource Identifiers (RRID)	Dilution
Calbindin	Mouse	Swant	300	AB_10000347	1:2000
Calbindin	Rabbit	Swant	CB38	AB_2721225	1:2000
Calretinin	Mouse	Swant	6B3	AB_10000320	1:2000
Calretinin	Rabbit	Swant	7699/4	AB_2313763	1:2000
α-CamKII	Mouse	Boehringer Mannheim	1,481,703	AB_2314079	1:400
CD44	Rat	Calbiochem	217,594	AB_211582	1:250
GABA	Rabbit	ImmunoStar (Diasorin Inc.)	20,094	AB_572234	1:1000
GAD67	Mouse	Millipore (Chemicon)	MAB5406	AB_2278725	1:2000
GFAP	Rabbit	Sigma	G-9269	AB_477035	1:500
EAAT2	Mouse	BD Biosciences	611,654	AB_399172	1:500
Iba-1	Goat	Abcam	ab5076	AB_2224402	1:1000
NeuN	Mouse	Millipore (Chemicon)	MAB377	AB_2298772	1:200
Parvalbumin	Mouse	Swant	PV235	AB_10000343	1:2000
Parvalbumin	Mouse	Sigma	P3088	AB_477329	1:4000
goat anti-mouse IgG, Biotinylated	Goat	Vector Laboratories	BA-9200	AB_2336171	1:200
goat anti-rabbit IgG, Biotinylated	Goat	Vector Laboratories	BA-1000	AB_2313606	1:200
goat anti-rat IgG, Biotinylated	Goat	Vector Laboratories	BA-9400	AB_2336202	1:200
horse anti-goat IgG, Biotinylated	Horse	Vector Laboratories	BA-9500	AB_2336123	1:200
horse anti-mouse IgG, Biotinylated	Horse	Vector Laboratories	BA-2000	AB_2313581	1:200

In short, sections were rinsed in 0.01 m PBS, pH 7.4, followed by 10% serum matching the species of the secondary antibody, 5% bovine serum albumin, and 0.1% Triton X-100 in 0.01 m PBS blocking solution for 1 h and incubated for 2 days at 4 °C in primary antibody. Some sections were double-labeled, while others were single-labeled. The sections were rinsed in PBS and incubated overnight at 4 °C with biotinylated goat anti-mouse and/or goat anti-rabbit and/or goat anti-rat secondary antibodies and thoroughly rinsed with PBS. An avidin–biotin–peroxidase kit was used to label CB-, PV-, or CR-expressing neurons with diaminobenzidine (Zymed Laboratories) for single-label experiments and an SG peroxidase kit was used to label cells with the immunohistochemistry chromogen SG (Vector Laboratories) in double-labeling experiments. Glial cells were labeled using avidin–biotin–peroxidase kit with diaminobenzidine (Zymed Laboratories). Control experiments with the omission of primary and/or secondary antibodies showed no nonspecific labeling, as previously reported [46, 47, 130]. Sections were mounted and coverslipped as described above. All sections were examined to ensure consistent, sufficient labeling for accurate quantification. Sections from cases were omitted from individual quantitative analyses if staining was inconsistent and caused concern about the accuracy of quantitative results.

We used archival processed non-human primate tissue (*Macaca mulatta*) to examine additional components of layer 1 in the primate cortex and to validate the results of the analysis of human LPFC. Tissue was labeled with Nissl and Gallyas stain, as described above. Sections were labeled through immunohistochemistry with antibodies against NeuN (an alternate method for labeling neurons, which specifically targets neuronal nuclei), gamma-Aminobutyric acid (GABA), glutamate decarboxylase (GAD67), PV, CB, CR, alpha subunit of calmodulin kinase II (α-CamKII), Iba-1, and glial fibrillary acidic protein (GFAP). All staining protocols have been previously described [46, 47, 130], and were similar to the processing of human tissue (above).

### Processing for electron microscopy

Tissue was processed for EM using a high-contrast method [133]. Sections processed for EM were adjacent to Nissl- and immunohistochemistry-labeled sections. Sections were washed in 0.1% PB and postfixed in 6% glutaraldehyde using a variable-wattage microwave to improve tissue penetration of fixative and other reagents. Sections were first rinsed in 0.1 M cacodylate buffer followed by 0.1% tannic acid prior to serial rinses in heavy metal solutions (1% osmium tetroxide with 1.5% potassium ferrocyanide, 0.1 g of thiocarbohydrazide, and finally 2% osmium tetroxide). Heavy metals impregnate lipid bilayers, creating contrast between membranes and other tissue components. Sections were washed with water, stained overnight in 1% uranyl acetate, and were finally stained with lead aspartate prior to dehydration in serial alcohols. Dehydrated tissue was cleared in propylene oxide and embedded in LX112 resin which was hardened between sheets of Aclar film at 60 °C for long-term storage.

We identified cortical gray matter in processed tissue using a dissecting microscope. ROIs containing gray matter were cut from the Aclar sheets and reembedded in LX112 resin blocks for sectioning at the ultramicrotome. 50 nm-thick sections were cut and collected on single-slot pioloform grids for imaging with a scanning electron microscope (SEM). 1 μm-thick (semi-thin) sections were cut and mounted on gelatin-coated slides and stained with toluidine blue powder in distilled water. Semi-thin sections were dried and then covered with toluidine blue solution for 1 min on a hot plate prior to being rinsed with water and coverslipped as described [129, 131, 133].

### Imaging and quantitative analysis

#### Light microscopy

Layer 1 was identified in stained sections using reference maps [6, 77, 100, 101, 123, 124]. Neurons were identified and quantified in Nissl-stained sections using a validated algorithm [46]. We quantified neuron density in multiple ROIs on one series of coronal sections per case. Sequential sections in children were on average 500 μm apart, while those in adults were on average 400 μm apart. We used an unbiased statistical sampling method to sample the areas of interest using commercially available software (StereoInvestigator, MicroBrightfield). The counting frame for these analyses was set to 150–180 μm with a height of 8 μm and grid spacing of 150–560 μm. The thickness of the section was measured at each counting site and a guard zone was set at the top of each section (2 μm). We calculated the density of neurons in layer 1 (neuron density, also referred to as the packing density) by dividing the estimated number of cells in layer 1 in each case by the measured volume of layer 1 in mm^3^. Quantification of immunolabeled tissue was performed at 400x, while Nissl-stained tissue was quantified at 1000x. Quantification of immunohistochemistry-labeled tissue sections was exhaustive, meaning that all counting frames were analyzed (counting grid and frame size were equal).

We measured the area fraction of neurons in high-resolution images of Nissl-stained tissue (thionin and toluidine blue) and the area fraction of axons in toluidine blue- and osmium-stained sections. Area fraction is a measure of the percent of the overall tissue surface area which contains axons or neurons. We used a consistent protocol to modify brightness and contrast of each image through adjustment of the levels of the histogram to maximize the black and white tonal range, while keeping the midtones unchanged (value 1.00). We thresholded images to increase the contrast between neurons, myelinated axons, and neuropil and to ensure reproducibility. Thresholding was performed using a standardized function within ImageJ that maintains the image moment, or weighted average of the intensity of the image pixels, in the whole image: this separates the image into meaningful gray-level classes. All background correction occurred automatically at image acquisition, and was maintained through the use of identical levels of luminescence for all image acquisitions. Standardized functions within ImageJ performed all measurements.

To place our findings within the context of postnatal maturation and myelination of frontal cortical layer 1 in the human brain we studied myelination patterns throughout the neurotypical lifespan using the classical atlas of cortical myelination by Kaes [62]. Kaes processed tissue using Weigart’s myelin stain (Max Wolter’s variant) in thinly cut tissue (under 50 μm) and examined 12 regions of the cortex from both hemispheres across 45 human brains, ranging from 3 months to 97 years old. His study focused on the thickness of the layers, defined by their myelin level, during postnatal development [92]. Comparison of the density of myelin in adults in Kaes’ material and our processed sections allowed us to match analyzed ROIs between datasets based on their similarity. Among Kaes’ ROIs, the anterior and posterior frontal lobes included the anterior and lateral prefrontal cortices, while Kaes’ Gyrus Fornicatus ROI is the cingulate cortex, including the ACC, in line with Kaes’ descriptions [62] and previous assessments [92].

We scanned the illustrations of columns from 31 brains (3 months - 65 years old, mean age = 27 years old; 7 female) from the atlas (original publication) at high resolution and used the scans to estimate the plot profiles of acquired cortical columns in ImageJ. Kaes’ published atlas used in this study is out of copyright and the book is in the public domain. We estimated the average gray level (optical density) of layer 1 in these columns using the acquired plot profile.

#### Electron microscopy

We acquired high-resolution images using a scanning electron microscope (Zeiss Gemini 300 with STEM detector, Atlas 5 software) at magnifications ranging from 2000x to 50,000x as previously described [133].

Maps of processed tissue labeled with toluidine blue were used to identify layer 1 in EM-processed tissue. We sampled grids in an unbiased way, similar to that described above and as shown in Fig. 1. We acquired all images at 30 nm resolution and performed all analyses of these images in Image J. We directly circled all myelinated axon profiles and obtained the measurement of the angle, major and minor diameter, surface area, and perimeter of each axon.

Axon segment trajectory measurements were acquired directly from the outlined profiles of axons analyzed in individual electron micrographs. For each outlined axon profile we estimated the major and minor diameter, aspect ratio, and the angle trajectory of the major diameter. Previous studies have successfully used this method to describe changes in axon trajectory in autism [133], and these measurements have been shown to correlate with the local trajectory of myelinated axons using 3D-reconstruction [129]. We processed the raw measurements of the axon segment angles following a previously reported protocol [133]. Briefly, we aligned the peak angle for each section to 90 degrees to account for bias from image acquisition, and we calculated the standard deviation of the angles for each case. We used the proportion of elongated axons (axons with aspect ratio > 3) as a weighting factor to produce the final reported standard deviation measurement. Axons with aspect ratio < 3 appeared more circular, and the angles of these axons were not a reliable indicator of the axon trajectory. Axons with aspect ratios > 3 were more elongated, and produced reliable measurements.

#### Statistics and cross-validation

All values are reported as the mean ± standard deviation (SD). To ensure that the results of these studies had statistical power, we selected a sampling fraction and studied a tissue volume that resulted in an error of under 10%, as described [44, 47, 129, 131, 133]. Data was statistically analyzed using ANCOVAs in SPSS (IBM) in order to determine correlations with age, cause of death, PMI, gender, and other identifying characteristics of our sample. The Levene statistical test, which assesses the homogeneity of variances between studied groups, was used to assess differences in variability between groups. Results were considered statistically significant with a *p*-value under 0.05.

Many independent experimental and analytical methods, ranging from light and electron microscopy, toluidine blue labeling, and myelin staining were used to cross-validate the results and minimize the effects of any potential experimental bias. We used archival monkey tissue to further validate our findings in human tissue, ensuring that factors in human tissue analysis and acquisition, such as PMI or quality of fixation, did not bias our conclusions. Additionally, multiple researchers analyzed subsets of the same data.

## Results

We studied excitatory and inhibitory elements in LPFC layer 1 in neurotypically developing children and adults with the aim of identifying age-related changes in the structure of layer 1. We compared normative changes with those seen in individuals with a diagnosis of autism in order to identify atypical age-related changes in network structure that may influence the development of autism symptomatology. To validate our findings in human tissue, we qualitatively examined layer 1 in the eulaminate LPFC and in the limbic-dysgranular ACC in human and non-human primate tissue to assess key characteristics of this layer that underlie its dynamic role in cortical development and function.

### Myelinated axons in layer 1: Normative development and changes in autism

**Fig. 2:**
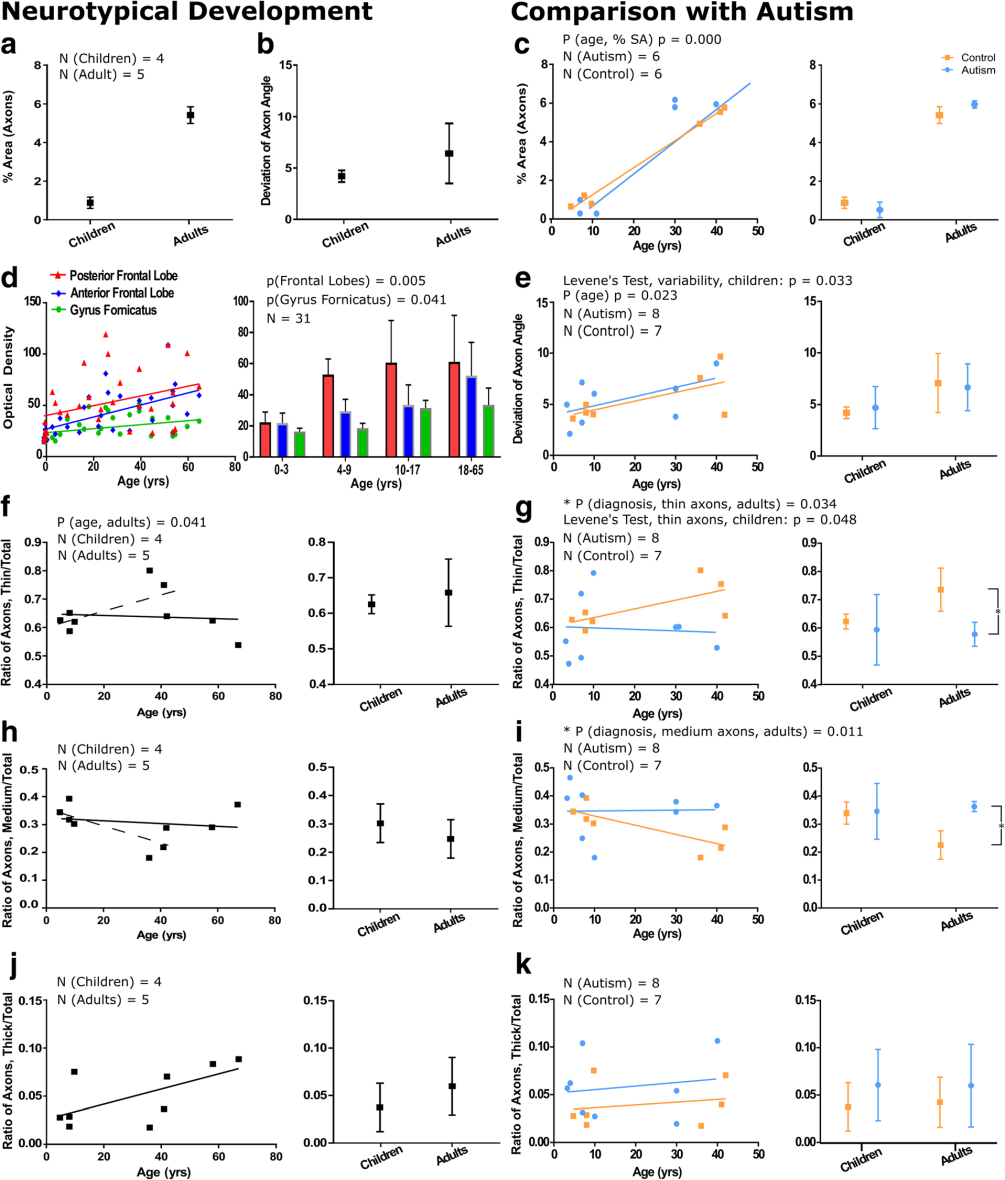
Myelinated axons in layer 1 of prefrontal cortex show significant changes in development and in autism. **a** The percent surface area occupied by axons in children is low (0.89 ± 0.29%), but increases significantly to 5.4 ± 0.44% in adults. **b** Axon trajectories are less variable in children than in adults in neurotypical development, suggesting increased myelination of diverse pathways in adulthood. **c** The percent surface area occupied by myelinated axons was similar in autism and neurotypical groups. The percent surface area occupied by axons increased significantly in adults from both groups (*p* = 0.000), shown on a case-by-case basis (left graph) and average by age group (right graph). **d** We analyzed images of layer 1 from the atlas of Kaes [62] to cross-validate our findings. Trends of myelin development in layer 1 of the anterior and posterior frontal lobes, which are similar to anterior and lateral prefrontal cortices, and Gyrus Fornicatus, which includes the anterior cingulate cortex, showed increases in myelination with age. In the posterior frontal lobe, layer 1 in children aged 0–3 was less myelinated than older children and adults. The anterior frontal lobe and anterior cingulate cortex had a more prolonged period of myelination and overall had a lower level of myelination compared with the posterior frontal lobe. **e** In children with autism there was a significant increase in the variability of axon trajectory heterogeneity when compared to neurotypical children (*p* = 0.033). In adults, this variability was similar between groups. **f** The relative proportion of thin axons remained approximately stable through neurotypical development. The solid line shows the overall stable trend, the dotted line shows the increase in the relative proportion of thin axons when comparing children and young adults. There was a trend towards a decline in the proportion of thin axons with age in adulthood. **g** The proportion of thin axons in neurotypical children was on average 64%; in children with autism, there was significant variation in the percentage of axons that were thin (range: 47–75%). Neurotypical adults had a higher average proportion of thin axons than adults with autism (mean control = 73.6 ± 7.7%, mean autism = 57.8 ± 4.1%, *p* = 0.034). **h** The relative proportion of medium-caliber axons remained approximately stable through neurotypical development. The solid line shows the overall stable trend, the dotted line shows the decrease in the relative proportion of medium-caliber axons when comparing children and young adults. There was a trend towards an increase in the proportion of medium-caliber axons with age in adulthood. **i** There was significant variability in the proportion of medium-caliber axons in children with autism. In adulthood, there was a significant increase in the relative proportion of medium-caliber axons in adults with autism compared with neurotypical adults (mean control = 22.5 ± 5.1%, mean autism = 36.2 ± 1.8%, *p* = 0.011). **j** There was a trend towards an increase in the relative proportion of thick axons in neurotypical development. **k** Developmental trends in the proportion of thick axons were not different between autism and control groups. Left panels in **e-k** present developmental trends on a case-by-case basis, and right panels show mean ± SD for children and adults with and without an autism diagnosis. Autism cases 4021, 4029, AN 03221, 5144, 1182, B-5173, B-6232, B-6677, and control cases 451, 4203, 4337, 3835, B-6004, B-5353, and B-4981 were used for electron microscopy comparisons. Control adults HAW and HAY were included in the study of neurotypical development and aging

**Fig. 3:**
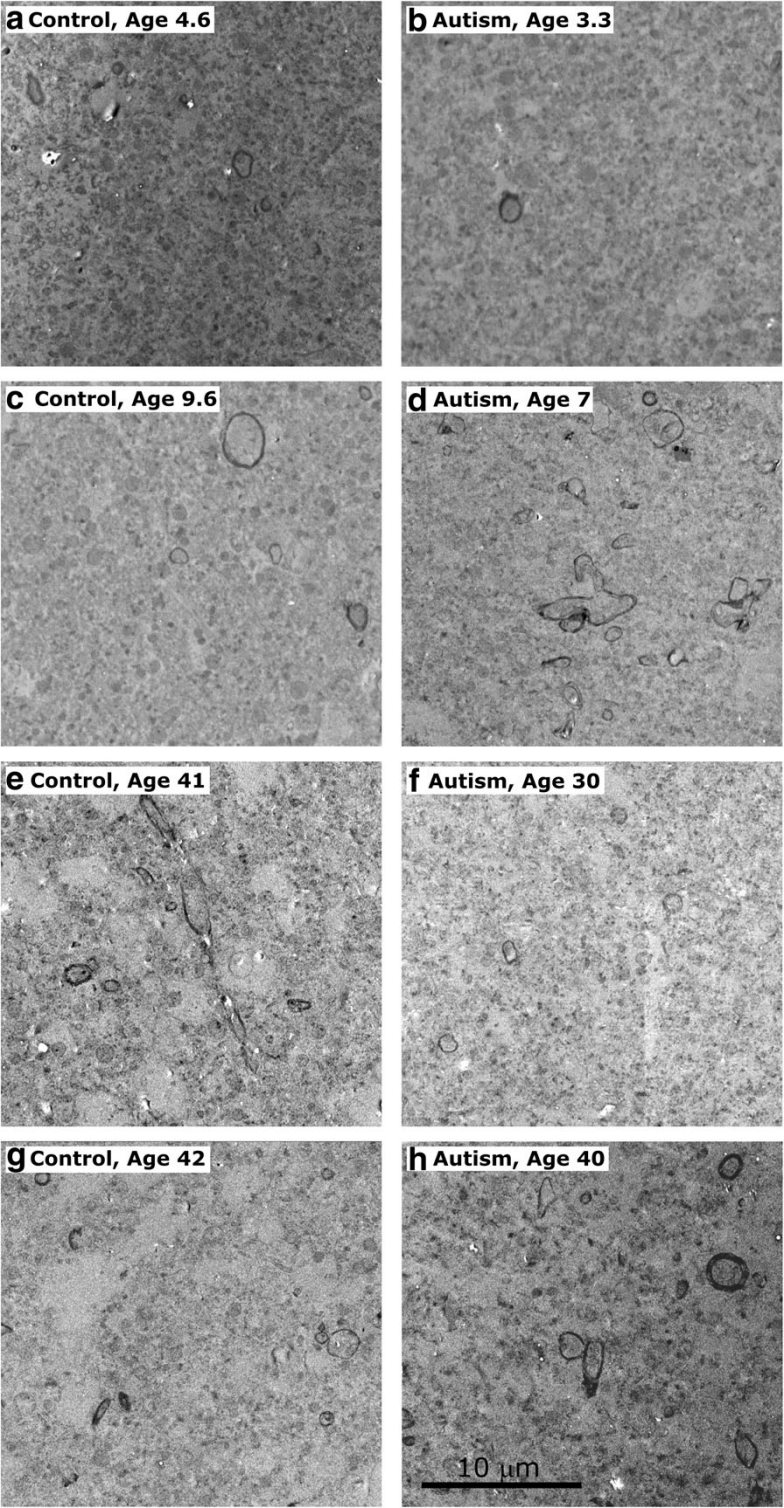
Myelinated axons in LPFC layer 1 analyzed at the electron microscope level in young children [cases 451 (**a**), 4021 (**b**)], older children [cases M3835 M (**c**), AN 03321 (**d**)], and adults [cases 5353 (**e**), 5173 (**f**), 4981(**g**), 6232 (**h**)] illustrate described trends. In younger children, who overall had fewer myelinated axons, there is still visible reduction in density of myelinated axons in individuals with autism (**b**). In older children there was a clear increase in the density of myelinated axons in autistic children (**d**), representing the variability inherent in autism, and multiple branching axons can be seen within the field. In adults, there was a decrease in proportion of small axons in individuals with autism with no visible change in overall axon density (*p* = 0.390) (**f, h**). Scale bar in (**h**) applies to all panels

We analyzed the structure and organization of myelinated axons in neurotypical individuals and compared normative developmental trends with the trends seen in individuals with autism (Fig. 2). In layer 1, myelinated axons derive from cortical and subcortical excitatory feedback pathways and local axons from excitatory and inhibitory neurons. Images of axons in representative children and adults with and without a diagnosis of autism are shown in Fig. 3. Analysis of neurotypical children and adults is shown in the left panels and a comparison with individuals with autism is in the right panels.

#### Normative development of myelinated axons in layer 1

We examined functionally relevant subpopulations of myelinated axons in layer 1 in neurotypically developing children and adults and assessed the increase in myelination using two complementary approaches. First, we measured the surface area occupied by myelinated axons using area fraction measures in osmicated and Nissl toluidine blue-stained sections. Myelinated axons occupied little of the surface area of layer 1 in childhood (mean ± SD: 0.89 ± 0.29%); this increased significantly (*p* = 0.000) to 5.4 ± 0.44% in adults (Fig. 2a). We also calculated the angle trajectories of all myelinated axon profiles, and we evaluated the organization of the network within layer 1 by determining the variability in the trajectories of the axons in all cases. Variability in myelinated axon trajectory deviation was small in childhood, ranging from 3.6^o^ - 5.0^o^ (4.20 ± 0.56^o^), and increased in adulthood (6.08 ± 2.60^o^), potentially reflecting the increased myelination of more diverse pathways with age (Fig. 2b).

Analysis of the laminar density of myelin in layer 1 of the anterior and posterior prefrontal cortex and the cingulate cortex (gyrus fornicatus) from cases used in Kaes’ atlas was in line with our quantitative analysis at the light and electron microscope. We observed a gradual and significant increase in myelination of layer 1 with age: anterior and posterior frontal regions had a steeper rate of increase in myelination during development than the cingulate cortex and higher levels of myelin in adulthood [p(Frontal Lobes) = 0.005; p(Gyrus Fornicatus) = 0.041] (Fig. 2d).

Axon caliber is correlated both with the length of the pathway and with efficiency of signal transduction: thick axons participate mainly in long-range connections and transmit signals faster than thin axons, which are mainly found in short-range connections [58, 131]. Therefore, we assessed the sizes of myelinated axons in layer 1 in order to identify potential changes in the relative proportion of different pathways and overall changes in the efficiency of signaling at different developmental stages. We divided axons into three categories: thin axons (diameter < 0.84 μm), medium-caliber axons (0.84 μm < diameter < 1.18 μm) and thick axons (diameter > 1.18 μm), as described [129, 131, 133]. In neurotypically developing children, 62.3 ± 2.6% of myelinated axons in layer 1 were thin while 33.9 ± 3.9% were of medium caliber; few thick myelinated axons were present (Fig. 2f, h, j). There were no significant differences in these proportions between children and adults. In adults there was an age-related decline in the relative proportion of thin myelinated axons and increase in the relative proportion of medium-caliber myelinated axons in layer 1 (*p* = 0.041), potentially relating to changes in pruning or branching characteristics in the neuropil that may occur with normal aging.

#### Changes in the myelinated axons of layer 1 in autism

We found significant changes in the organization and structure of myelinated axons in layer 1 of both children and adults with autism when compared with neurotypical individuals. In order to ensure appropriate age-matching of subjects, we excluded unmatched older control adults from comparisons.

The density of myelinated axons increased with age in individuals with autism and controls (Fig. 2a, c) (*p* = 0.000; no difference between autism and control, *p* = 0.349). The variability in myelinated axon trajectory deviation was significantly larger in children with autism compared to neurotypical children (*p* = 0.033): this difference did not persist in adults, although there was a significant increase in myelinated axon trajectory deviation with age (*p* = 0.023) (Fig. 2e). This finding suggests that there is significant local circuit disorganization in individuals with autism beginning early in postnatal development. In children with autism, we found significantly increased variability in the proportion of myelinated thin axons (SD Autism = 12.5%, SD Control = 2.6%, *p* = 0.048) (Fig. 2g). In adults with autism, the proportion of myelinated thin axons in layer 1 was significantly lower than that in controls (mean Control = 73.6 ± 7.7%, mean Autism = 57.9 ± 4.1%, *p* = 0.034) (Fig. 2g), accompanied by a significant increase in the proportion of myelinated medium-caliber axons (mean Control = 22.5 ± 5.1%, mean Autism = 36.2 ± 1.8%, *p* = 0.011) (Fig. 2i). These changes were not associated with parallel alterations in the density of axons in adults with autism (*p* = 0.390).

### Neuron population in layer 1: Normative development and comparison with autism

**Fig. 4:**
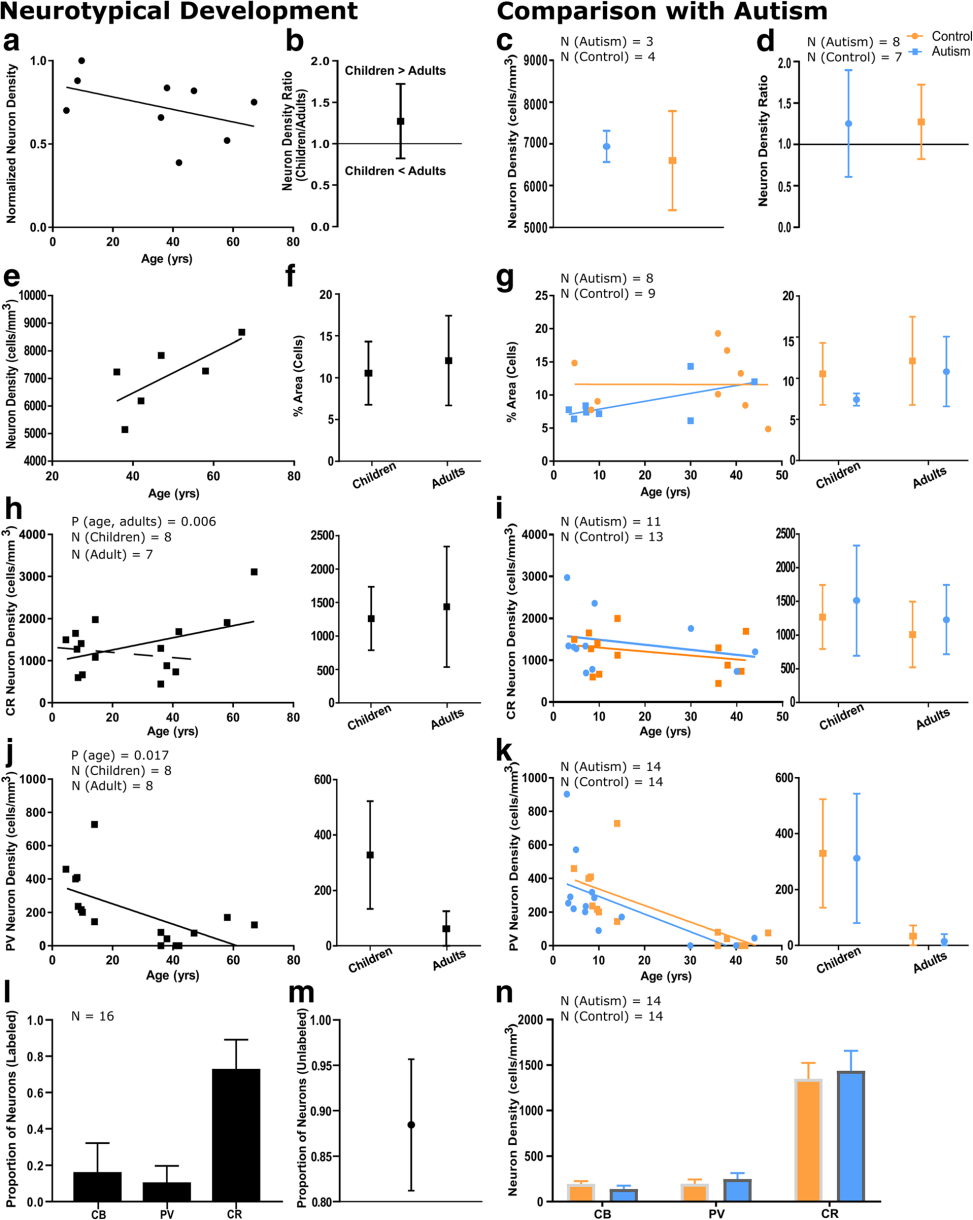
Neuronal populations in LPFC layer 1 change in parallel between children and adults with and without autism. **a** There was a trend towards a reduction in neuron density between neurotypical children and adults. Neuron density values were normalized such that the highest density has a value of 1. Cases used in this analysis: 451, 4337, M3835 M, 6004, 4981, HAW, HAY, HCD, HCF. **b** There was a reduction in neuron density between typical children and adults, with a ratio of mean neuron density in children/adults of approximately 1.27. **c** Mean neuron density in adults with and without autism (mean control = 6602 ± 1186 cells/mm^3^, mean autism = 6939 ± 217 neurons/mm^3^) was not statistically different. **d** The decrease in neuron density from childhood to adulthood was not different between groups. **e** There was a trend towards an increase in neuron density with age between young and older adults. **f** The area fraction containing cells increased slightly in neurotypical development, likely representing simultaneous dilution of neurons within the neuropil and expansion of glial cell populations with age. **g** There was no difference between the percent surface area occupied by cells between autistic and neurotypical groups. **h** CR neuron density increased between children and adults. In this sample, this trend was primarily driven by high density of CR neurons in older adults. The solid line shows the overall trend towards an increase in CR neuron density with age, the dotted line shows the decrease in CR neuron density when comparing children and young adults **i** The trend towards a decrease in CR neuron density in layer 1 was similar in neurotypical individuals and individuals with autism. **j** PV neurons were present in layer 1 in neurotypical children; their density decreased significantly in adults, and in many adult cases these neurons were not found. **k** PV neurons were prominent in layer 1 of non-autistic (mean = 329 ± 69 neurons/mm^3^) and autistic children (mean = 312 ± 231 neurons/mm^3^), and their density decreased significantly in adults (mean control = 33 ± 39 cells/mm^3^, mean autism =15 ± 26 neurons/mm^3^, *p* = 0.006). **l** We calculated the relative proportions of the three inhibitory interneuron subtypes in layer 1. CR was the most prominent inhibitory interneuron subtype, labeling 73.07 ± 16.07% of labeled neurons. CB was expressed by 16.32 ± 15.81% of labeled inhibitory neurons, while PV was the least prominent interneuron subtype labeling only 10.60 ± 9.10% of labeled interneurons in layer 1. **m** 88.44 ± 7.24% of neurons were not labeled with calcium binding proteins in layer 1. **n** We calculated mean inhibitory neuron densities, including cases of all ages. CR-immunoreactive neurons were the densest neuron population (mean control = 1385 ± 696 neurons/mm^3^, mean autism = 1436 ± 733 neurons/mm^3^), followed by CB- (mean control = 194 ± 127 neurons/mm^3^, mean autism = 138 ± 124 neurons/mm^3^) and PV- neurons (mean control = 220 ± 215 neurons/mm^3^, mean autism = 248 ± 239 neurons/mm^3^). There were no significant differences between groups. Autism cases AN 03345, 1182, AN 04682, and B-6677 and control case HCF were not included in CR quantitative analyses due to inconsistent staining. Autism case B-6677 was not included in PV quantitative analyses due to inconsistent staining. Left panels in **g-k** present developmental trends on a case-by-case basis, and right panels show mean ± SD for children and adults

Next, we examined the overall neuronal population of layer 1 along with three functionally distinct subclasses of neurons labeled by the calcium-binding proteins PV, CB, and CR. In humans, neurons in layer 1 are overwhelmingly inhibitory: the vast majority of neurons in layer 1 express GABA [55] and exhibit inhibitory activity [106]. We characterized age-related changes in the density and distribution of neurons in layer 1 in neurotypical children and adults and compared them with trends seen in individuals with autism to identify whether changes in neuronal populations could be additional sources of excitatory-inhibitory imbalance in autism (Fig. 4).

#### Normative development of neurons in layer 1

We measured neuron density in layer 1 in neurotypically developing children and adults in Nissl-stained tissue. In order to ensure accuracy, tissue was oversampled to account for the low density and anisotropic distribution of cells in layer 1. Overall neuron density in layer 1 in adults was 7059 ± 1242 cells/mm^3^. Neuron density declined from childhood to adulthood (Fig. 4a, b), likely due to the increase in neuropil volume associated with branching and myelination during development. There was a trend towards an increase in neuron density in layer 1 with age in older adults (Fig. 4e), potentially relating to the changes in axon pruning with age described above. In children, we saw significant variability in the neuron density in layer 1, in line with reported heterogeneity in the maturation of the cortex [127]. Cells occupied 10.6 ± 3.8% of the area of layer 1 in children; this increased moderately to 12.1 ± 5.4% in adults (Fig. 4f).

#### Inhibitory neurons in layer 1 during neurotypical development

In order to further study this population of inhibitory neurons, we labeled tissue with the three calcium-binding proteins (PV, CB, and CR). The most prevalent inhibitory interneuron subtype in layer 1 in neurotypically developing individuals were CR+ interneurons (54–94% of labeled neurons) (Fig. 4l), which, in the upper cortical layers, primarily serve a disinhibitory role through selective targeting of CB+ interneurons [29, 86]. This is in line with previously reported findings in layer 1 in the adult human and non-human primate cortex, which has been shown to predominately contain CR neurons, including remnants of the Cajal-Retzius cell population [40, 81, 82]. There were also few calbindin-expressing neurons (mean = 194 ± 127 cells/mm^3^), which did not vary significantly with age. In children, CR neurons had a mean density of 1270 ± 475 cells/mm^3^, which increased in adults to 1437 ± 902 cells/mm^3^ (Fig. 4h). We separately analyzed younger (37–47 years old) and older adults (58–67 years old) to identify possible age-related changes in CR neuron density. In younger adults CR neuron density in layer 1 was 1009 ± 488 cells/mm^3^, while preliminary data from two older adults suggests an increase in CR neuron density (mean = 2508 ± 847 cells/mm^3^, *p* = 0.006). Quantification of additional cases with ages over 60 years would be necessary to confirm this change. Of note, the majority of neurons in layer 1 in adults were not labeled by calcium-binding proteins (69–94%) (Fig. 4m).

#### PV-immunolabeled neurons in layer 1 of children and adolescents

Our results show for the first time to the best of our knowledge that neurotypically developing children and adolescents have a population of PV neurons in layer 1 in LPFC (mean = 329 ± 194 neurons/mm^3^) (Fig. 4j). Previous studies have identified PV-expressing Cajal-Retzius and non-Cajal-Retzius neurons in layer 1 of prenatal and newborn humans [30, 121]: the neurons identified in this study may be members of these neuronal populations, which persist later in development than previously believed. Further studies are needed to identify the functional role of these PV-expressing neurons of layer 1, which may or may not serve the same strongly-inhibitory role of the PV-expressing neurons of the deeper cortical layers.

There was a significant age-related reduction in the density of PV interneurons in adults, with many cases having no detectable PV neurons in layer 1 (mean = 62 ± 22 neurons/mm^3^, *p* = 0.017) (Fig. 4j). Younger adults had a mean PV neuron density of 33 ± 39 cells/mm^3^, which increased to 148 ± 32 cells/mm^3^ in older adults, based on preliminary data.

#### No changes in neurons in layer 1 in autism

There was no significant difference between control and autism groups in overall neuron density in layer 1 in adults (mean Autism = 6939 ± 217 neurons/mm^3^; mean Control = 6602 ± 1186 cells/mm^3^, *p* = 0.453) (Fig. 4c). The decline in neuron density associated with age (Fig. 4d) and the percent surface area occupied by cells (Fig. 4g) were also similar between groups. Previous reports have identified as much as a 30% increase in neuronal density in the prefrontal cortex in autism [3, 16]; our findings suggest that these increases are not due to changes in layer 1.

We assessed the densities of the three interneuron subtypes in layer 1 of LPFC in individuals with autism and found no differences from controls [p(CR, Autism vs Control) = 0.470, p(PV, Autism vs Control) = 0.828, p(CB, Autism vs Control) = 0.711] (Fig. 4i, k, n). This suggests that the changes reported above in myelinated axon structure in autism are not due to changes in the cellular populations intrinsic to layer 1, but instead are due to changes in branching and maturation of local networks or incoming pathways.

### Cross-validation of layer 1 features through systematic comparison of frontal cortices in adult humans and in a non-human primate animal model

**Fig. 5:**
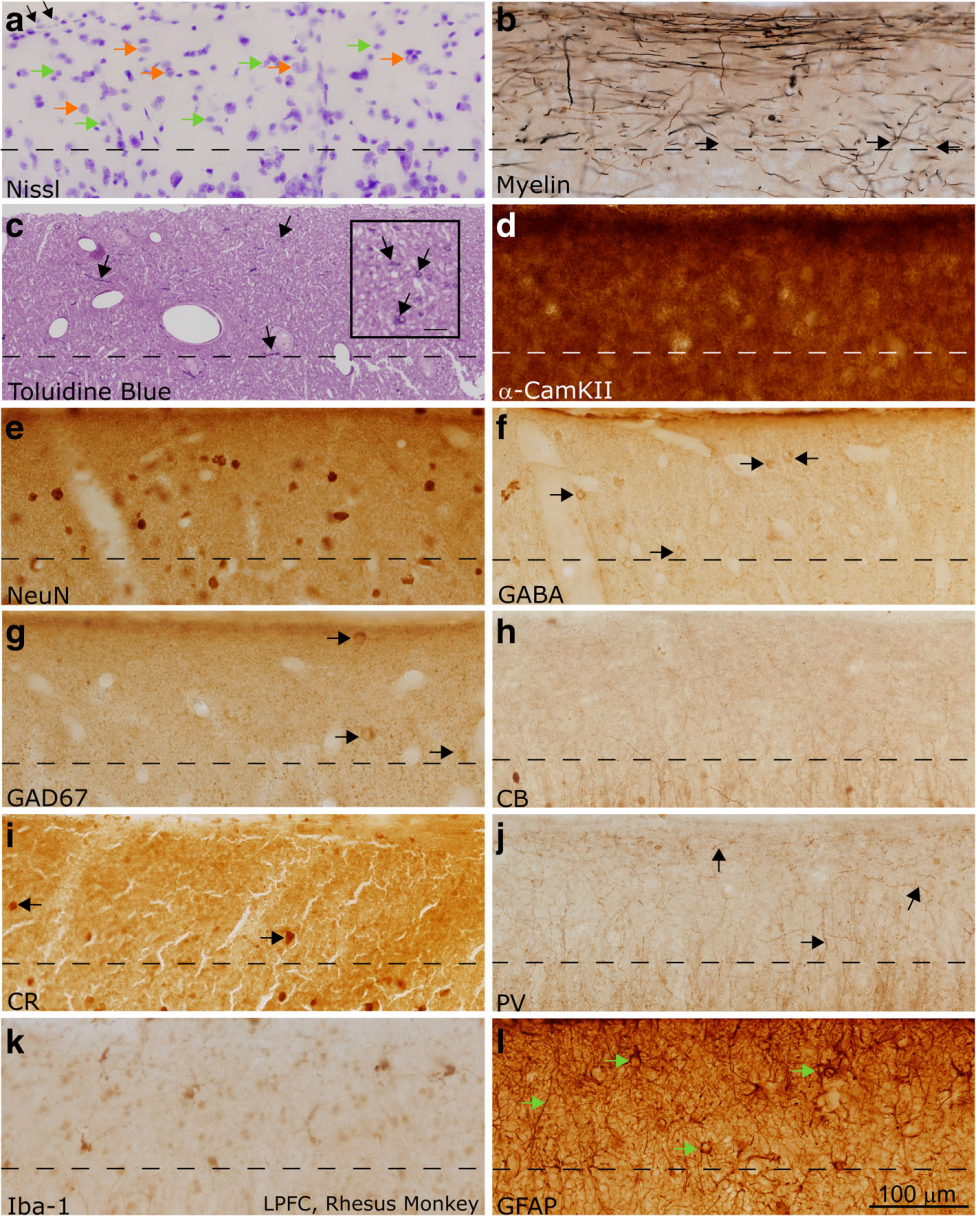
Layer 1 in the LPFC of the adult non-human primate (rhesus macaque). **a** Section labeled with Nissl showed a moderate density of neurons and glia, mainly astrocytes, in layer 1 (examples of neurons are marked with orange arrows, glial cells are marked with green arrows). Superficially, the glia limitans was visible as a dense band of astrocytes (shown with black arrows). **b** Myelin (Gallyas) stain showed the dense band of myelinated axons within superficial layer 1. Myelinated axons were seen penetrating layer 1 with diverse trajectories prior to joining the myelinated superficial plexus (black arrows). These axons came from long-range pathways or local interneurons. **c** Toluidine blue labeling of a 1 μm thick section from osmicated tissue revealed Nissl-stained cells and their processes, including profiles of myelinated axons (shown with black arrows). There was a higher density of thin axon profiles in superficial layer 1, in line with myelin stain shown in (**b**). Scale bar in inset measures 10 μm. **d** α-CamKII is a marker of synaptic turnover, and is used to identify areas of high plasticity. It labeled a dense population of processes in layer 1. **e** NeuN labels most neuronal nuclei, and in our material all layer 1 neurons that were labeled with Nissl were also labeled with NeuN (not shown). NeuN labeling clearly showed that the density of labeled neurons in layer 1 was low, and the majority of the cellular density in layer 1, as seen in (**a**), was not due to neurons but instead could be attributed to glia. **f-g** GABA (**f**) and GAD67 (**g**) label inhibitory neurons in the cortex (labeled with black arrows). There was a low density of labeling in both sections. Comparison with sections stained with NeuN (**c**) suggested that many neurons in layer 1 did not express GABA and its synthesizing enzymes strongly. **h-j** CB, CR, and PV labeled subpopulations of inhibitory interneurons. CB (**h**) and PV (**j**) did not label cell bodies in layer 1 in the adult non-human primate, while CR (**i**) labeled few cell bodies (shown with black arrows). PV (**j**) labeled a population of axons which joined the superficial plexus, and may represent either thalamocortical axons or axons of local inhibitory interneurons (shown with black arrows). CB (**h**) and CR (**i**) also labeled few axons in layer 1, but were not as visible as the PV-labeled processes. **k** Microglia with various morphologies, labeled with Iba-1, could be seen in layer 1. **l** GFAP labeled the cell bodies and dense processes of astrocytes within layer 1 (shown with green arrows). Images were acquired such that the top edge of the images underlie the pia. Dotted lines indicate the border with layer 2 in all panels. Calibration bar in (**l**) applies to all panels

**Fig. 6:**
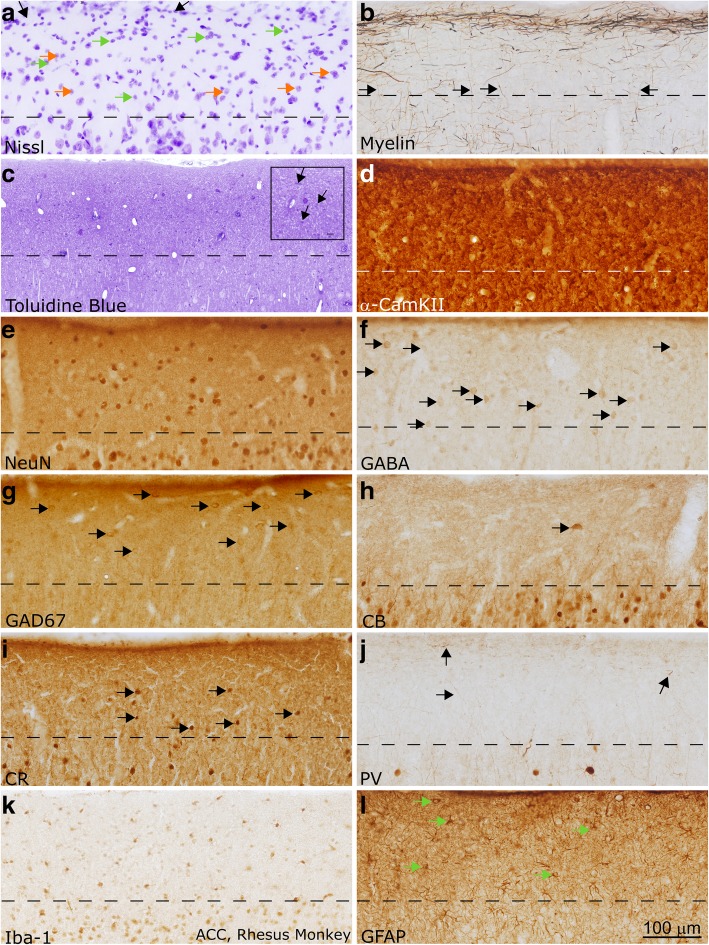
Layer 1 in the ACC of the adult non-human primate (rhesus macaque). **a** Section labeled with Nissl showed a moderate density of neurons and glia, mainly astrocytes, in layer 1 (examples of neurons are marked with orange arrows, glial cells are marked with green arrows). Superficially, the glia limitans was visible as a dense band of astrocytes (shown with black arrows). **b** Myelin (Gallyas) stain showed the relatively light band of myelinated axons within superficial layer 1 in ACC. This band was lighter than that seen in LPFC, following overall trends in myelination between those areas. Black arrows show axons entering layer 1 from layer 2. **c** Toluidine blue labeling of a 1 μm thick section from osmicated tissue revealed Nissl-stained cells and their processes, including profiles of myelinated axons (shown with black arrows in inset). There was a low density of axon profiles in superficial layer 1, in line with the light myelin stain shown in (**b**). Scale bar in inset measures 10 μm. **d** α-CamKII is a marker of synaptic plasticity. It labeled a dense population of processes in layer 1. **e** NeuN labeled all layer 1 neurons that were labeled with Nissl (not shown). NeuN labeling clearly showed that the density of labeled neurons in layer 1 was low, and the majority of the cellular density in layer 1, as seen in (**a**), was not due to neurons but instead could be attributed to glia. **f-g** GABA (**f**) and GAD67 (**g**) labeled inhibitory neurons in the cortex (labeled with black arrows). There was a moderate density of labeling in both sections, comparable to what was seen in the NeuN stain (**c**). **h-j** CB, CR, and PV labeled subpopulations of inhibitory interneurons. CB (**h**) and PV (**j**) labeled primarily neuron processes in layer 1 in the adult non-human primate, while CR (**i**) labeled few cell bodies (shown with black arrows). Black arrows in (**j**) show PV-labeled axons. **k** Microglia with various morphologies, labeled with Iba-1, could be seen in layer 1. **l** GFAP labeled the cell bodies and dense processes of astrocytes within layer 1 (shown with green arrows). Images were acquired such that the top edge of the images underlie the pia. Dotted lines indicate the border with layer 2 in all panels. Calibration bar in (**l**) applies to all panels

**Fig. 7:**
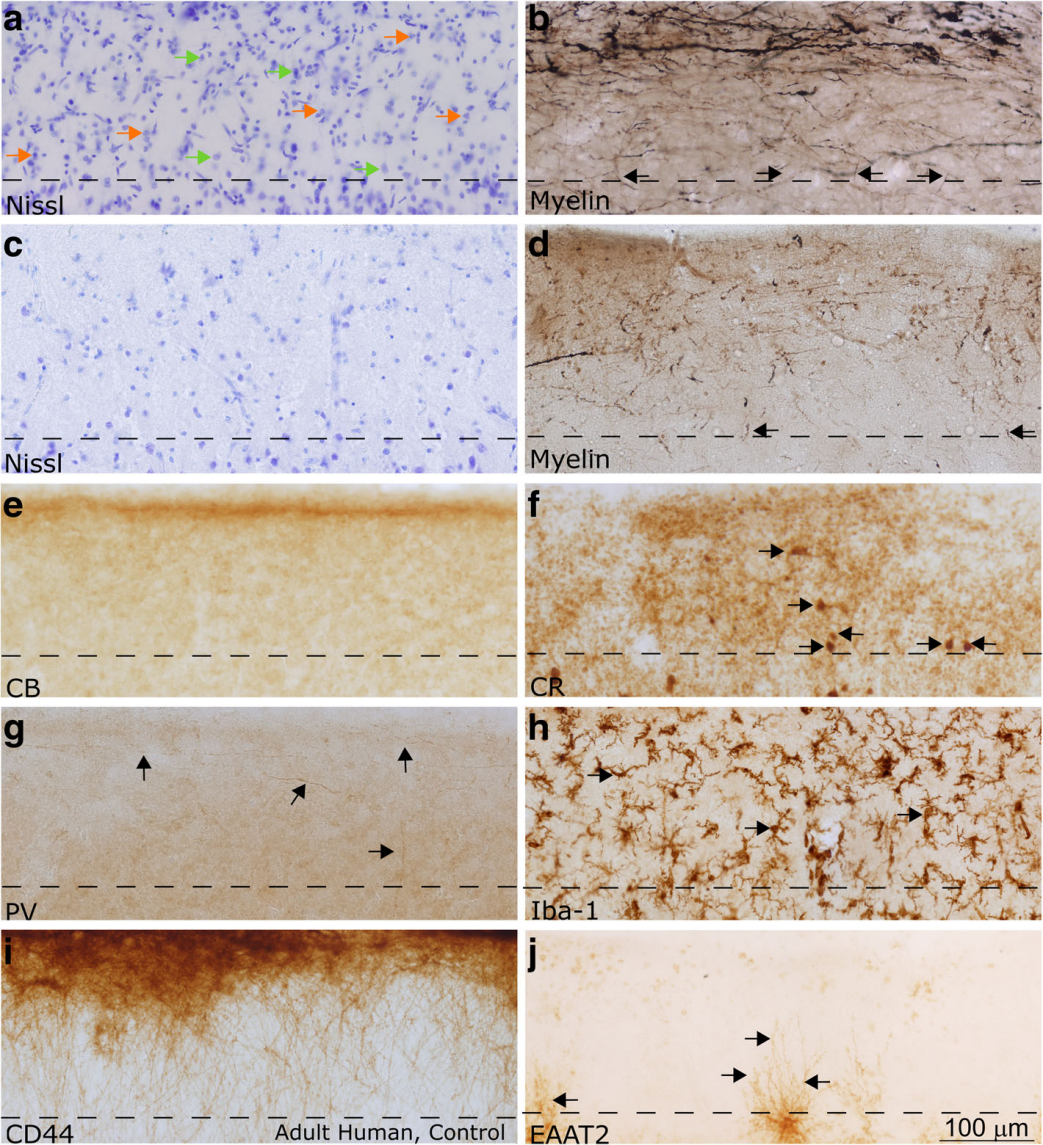
Layer 1 in the LPFC and ACC of the adult human. All images in this figure were acquired from neurotypical adult cases (HCD, HCF, HAW, HAY). **a-d** Nissl (**a**) and myelin (**b**) in the LPFC showed stark differences from ACC (**c-d**). ACC had a thicker layer 1, and reduced density and thickness of myelinated axons in layer 1. Permeating blood vessels and endothelial cells were also visible in the Nissl-labeled section from LPFC layer 1 in (**a**). Examples of neurons in (**a**) are marked with orange arrows, glial cells are marked with green arrows. Myelin (Gallyas) stained tissue (**b, d)** showed a dense plexus of myelinated axons in superficial layer 1, consistent with observations in the non-human primate. While the majority of axons were horizontal, some also had diagonal trajectories, consistent with axons from incoming pathways (black arrows). **e-g** CB, CR, and PV labeled inhibitory interneuron classes. CB (**e**) labeled scant processes in layer 1 (labeled with black arrows), while CR (**f**) labeled a low density of neurons and few processes in layer 1 (labeled with black arrows). PV (**g**) labeled processes that joined the plexus of axons in superficial layer 1 (labeled with black arrows). **h** Iba-1 labeled microglia within the cortex, including layer 1 (examples of microglia are labeled with black arrows). Superficial microglia extended processes mostly parallel to the pial surface, while microglia deeper in layer 1 had processes oriented in multiple directions. **i** CD44 labeled interlaminar astrocytes, which sat on the pial surface and sent processes towards layer 2. These astrocytes are exclusive to higher-order primates. **j** Astrocytes labeled with excitatory amino acid transporter (EAAT2) were not present in layer 1; however, the processes of labeled astrocytes from layer 2 penetrated into the deep part of layer 1 (labeled with black arrows). Images were acquired such that the top edge of the images underlie the pia. Dotted lines indicate the border with layer 2 in all panels. Calibration bar in (**j**) applies to all panels

**Fig. 8:**
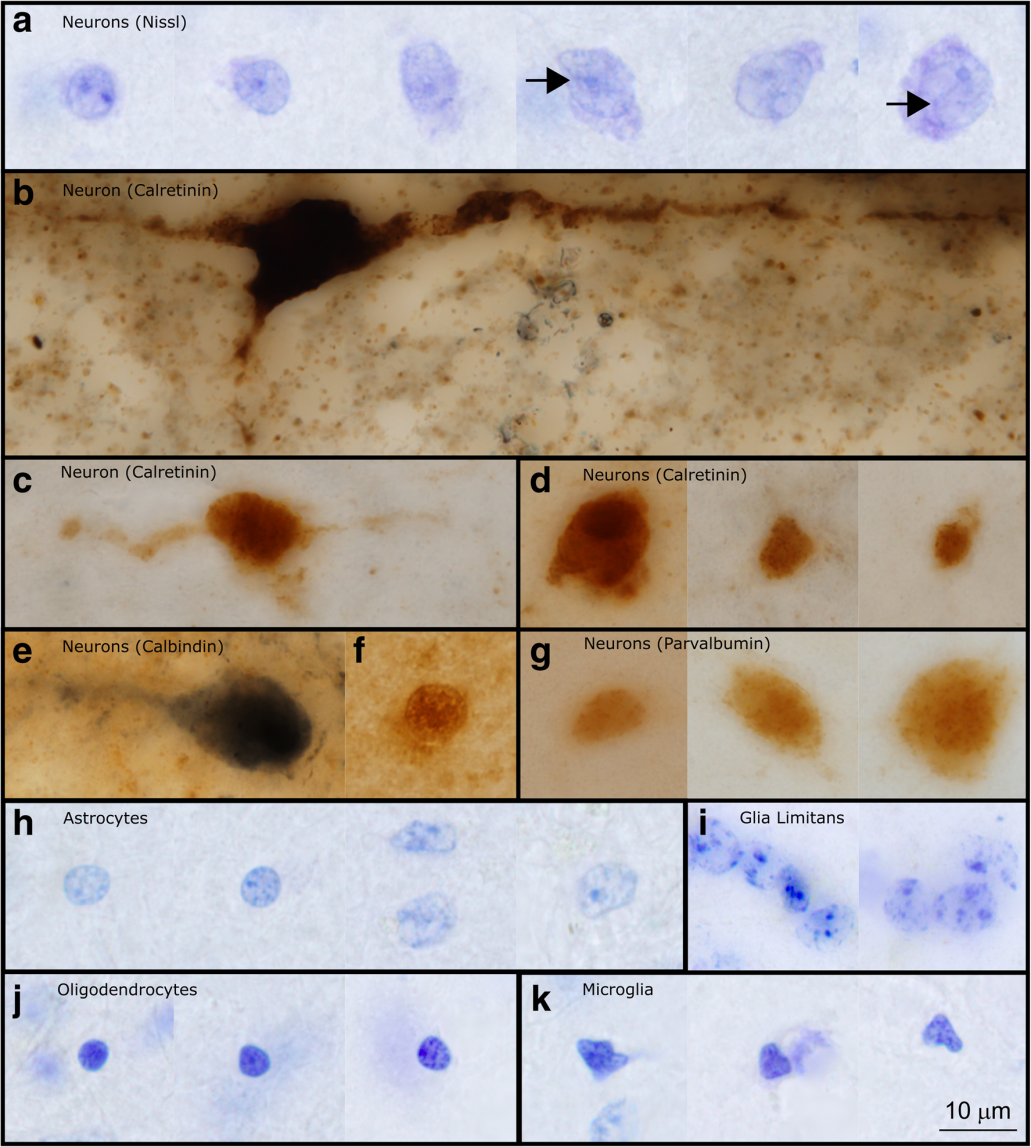
High magnification images of representative neurons and glia in layer 1 of the LPFC in the human brain. **a** Neurons in layer 1 had a broad range of sizes. In Nissl preparations it was possible to see the nucleus, nucleolus, and a rim of cytoplasm around labeled neurons. In larger neurons it was possible to observe folding of the nucleus (shown with black arrows in figure) (Case HCD). **b** This large, subpial neuron labeled with CR is likely a Cajal-Retzius cell. This image shows its large cell body and horizontal extensions, along with a descending process which is typical of Cajal-Retzius cells (Case AN4722). **c-d** CR labeled neurons of multiple sizes and shapes were found in layer 1 of the human cerebral cortex. CR-labeled neurons in layer 1 had multiple orientations within layer 1, including horizontally oriented cells (**c**) and cells with more typical interneuron morphology (**d**). **e-f** CB-labeled neurons were also diverse in shape and size. These neurons may also be oriented horizontally (**e**) (Case 4722, Case 3835). **g** Representative PV-labeled neurons in layer 1 (Case 4337). **h-i** Astrocytes in layer 1 had multiple morphologies. The astrocytes of the glial limitans were compact and formed tight groups (**i**) (Case HCD). **j** Three oligodendrocytes in human layer 1 (Case HCD). Oligodendrocytes in human tissue had varied staining characteristics: they could be darkly labeled, as seen in the first two images, or lightly labeled, as shown in the third image. **k** Microglia in layer 1 had irregularly shaped nuclei, distinguishing them from darkly stained oligodendrocytes (Case HCD). Calibration bar in (**k**) applies to all panels

In order to confirm that our results were not influenced by post-mortem factors associated with the acquisition and processing of human LPFC tissue, we cross-validated our findings with a qualitative assessment of the cellular populations and myelination in another prefrontal region of the human brain, the ACC, and in optimally-fixed rhesus macaque tissue. A comparison of the patterns of immunohistochemical labeling of immersion-fixed brain tissue with a post-mortem delay and ideally-fixed, perfused brain tissue has suggested that the detection of some calcium-binding proteins is susceptible to post-mortem changes [50]. As both the gray and white matter of the non-human primate have significant structural and organizational similarity to that of the human [131], we used perfused tissue from rhesus macaque monkeys to identify the key structural characteristics of the normal layer 1 and to confirm that our findings in the human were not attributable to changes in immunoreactivity due to post-mortem factors. We therefore compared the structure of layer 1 in LPFC to ACC in both species and identified conserved and altered features of layer 1 in these structurally distinct areas (Figs. 5, 6 and 7). Images of representative neurons and glial cells are summarized in Fig. 8. Our comparisons, summarized below, highlight the high biological and statistical significance of the reported changes in both typical and atypical postnatal development of layer 1 in the human LPFC.

There was a moderate density of neurons in layer 1 in both areas (Fig. 5a, e; Fig. 6a, e; 7a, c). A subpopulation of layer 1 neurons was labeled with GABA or GAD67, indicating that they were inhibitory (Figs. 5f-g, 6f-g). Few neurons were labeled by CR, which indicates that they were either inhibitory or non-GABAergic Cajal-Retzius neurons. CB- and PV-expressing inhibitory neurons were scarce in layer 1 of adult primates, although some cell processes within layer 1 expressed these markers strongly (Figs. 5h-j, 6h-j, 7e-g). Qualitative observations from the non-human primate were in line with qualitative and quantitative findings in human subjects. These observations suggest that post-mortem factors did not significantly alter the conclusions of our immunohistochemical analysis.

Varied populations of glia were present in layer 1 (Figs. 5k-l, 6k-l, 7h-j). Astrocytes, which participate in the regulation of neuronal signaling [105], were highly immunoreactive for GFAP, a glial structural protein associated with astrocyte activation (Figs. 5l, 6l), but did not express EAAT2, an excitatory amino acid transporter responsible for the reuptake of glutamate (Fig. 7j). We furthermore identified interlaminar astrocytes, which are typical of layer 1 in the primate brain and extend processes though layers 2 and 3 [19, 21, 79, 80] and marginal astrocytes on the pial border that extended processes towards layer 2 (Fig. 7i). The unique structure and function of astrocytes in layer 1 likely affects the regulation of signaling in this layer and merits future consideration.

There was a dense, superficial plexus of myelinated axons in layer 1. Myelinated axons were seen penetrating layer 1 to join this plexus (Figs. 5b, 6b, 7b, d), supporting the assertion that some myelinated axons within layer 1 originate outside of this layer. This plexus was denser in LPFC than in ACC (Figs. 5b, 6b, 7b, d), reflecting an overall difference in myelination between these areas. The myelinated plexus in LPFC furthermore contained a higher density of PV-positive axons (Figs. 5j, 6j, 7g), deriving either from local interneurons or thalamocortical pathways [47]. Neuropil in layer 1 was densely labeled by α-CamKII (Figs. 5d, 6d), a marker of synaptic plasticity [75]: high levels of this protein in layer 1 suggests that networks within layer 1 in these cortices are remarkably plastic. Differences between the structure of axon networks in the granular LPFC and limbic ACC despite relative homogeneity in the cellular populations of layer 1 supports our conclusion that the plastic and variable axonal networks within layer 1 could be a target of dysfunction in autism.

## Discussion

We present evidence of postnatal changes in the balance of excitation-inhibition in the maturing prefrontal cortex throughout typical development and in autism, using a large cohort of human subjects at a variety of postnatal ages. Our findings reveal specific changes in the structure of pathways and cellular populations within layer 1 of the LPFC through typical development. We also present evidence suggesting that atypical, age-associated changes in the organization and relationship between pathways and cellular populations in layer 1 of the LPFC may underlie the dysfunctional balance of excitation-inhibition in the maturing prefrontal cortex in autism.

In typical postnatal development, the density of myelinated axons in layer 1 of the prefrontal cortex increased with age, in line with previous studies on the maturation of white matter pathways [74, 89]. Specifically, in LPFC, the relative proportion of thin myelinated axons in layer 1 of adults was significantly higher than what has been previously described in the white matter, where thin axons represented 36% of the myelinated axon population [129, 131, 133]. This supports our understanding that axons enter the cortex and quickly branch, resulting in an overrepresentation of thin axons in superficial cortical layers. The increased density of thin axons in layer 1 was accompanied by an increase in the volume of neuropil, including an increase in unmyelinated axon density, estimated by the gray level index, and a decrease in neuron density in adults. We noted both an increase in neuron density and a decrease in the proportion of thin axons in two older adults (ages 58 and 67), suggesting that reductive changes in neuropil structure, potentially due to pruning, may be associated with normal aging.

Surprisingly, a significant proportion of neurons in layer 1 did not express the calcium-binding proteins that are typically used as markers of inhibitory interneurons in the cortex of primates, even though close to 90% of neurons in layer 1 are GABAergic [41, 42, 54–56]. Similar staining patterns in human and optimally-fixed rhesus macaque brain tissue corroborated this observation. These findings suggest that there are potential differences in the origin and physiological characteristics of interneurons in layer 1 when compared to other cortical layers, which may influence the regulation of GABAergic signaling in layer 1 [28, 102]. The emerging complexity of this layer in our study is in line with recent work that has led to the identification of novel neuronal types in layer 1 of humans [11]. The differences that we have identified in the expression of calcium-binding proteins between childhood and adulthood further suggest that the cellular composition of layer 1 changes significantly during postnatal development. We detected a significant population of PV-immunoreactive neurons in LPFC layer 1 of neurotypical children and adolescents, previously seen only pre- and peri-natally [30, 121]. The density of layer 1 PV-immunoreactive neurons decreased with age to negligible numbers in adults, suggesting developmental changes in the calcium dynamics, and therefore in synaptic speed and strength, of inhibitory neurons in layer 1 during development. This could have an effect on the processing of incoming feedback or neuromodulatory signals in childhood and adulthood, leading to differences in the balance of excitation-inhibition in the cortex.

We found significant evidence of disorganization of axon networks within layer 1 in individuals with autism. There was increased heterogeneity in the trajectories and the proportion of myelinated axons that were thin in children with autism compared with controls. While there was an increase in the ratio of thin myelinated axons in layer 1 in neurotypical adults, thin axon ratios did not change with age in autism, leading to a significantly lower proportion of thin myelinated axons in adults with autism compared to the control group. This suggests that layer 1 thin axon networks in adults with autism remained on average at similar levels as immature, less myelinated networks in childhood, in line with previous work that has shown increased axon branching at the border of the gray and white matter below interlinked medial and lateral prefrontal cortices in these and other cases [35, 111, 129–131, 133]. Increased axon branching and a decrease in the proportion of thin myelinated axons may be due to an increase in the density of unmyelinated axons, which we could not directly assess in this study. Future studies would need to assess changes in the qualities of the neuropil through development and in autism to confirm this hypothesis.

Alterations in axon structure may have broader implications for the efficacy of signal transmission in frontal cortical networks of individuals with autism. In particular, changes in axon caliber and the relative ratios of myelinated axon sizes directly influence the strength and persistence of the action potential generated by projection neurons, as well as their firing rate [58, 96], which play a key role in sustained activity during working memory and attentional tasks in LPFC [22, 65, 95, 99]. These, in turn, can underlie changes in the spread of activity and oscillations [12, 59, 67, 68, 73], and may be linked with changes in the expression of neuronal ion channels and regulatory proteins in individuals with autism [25, 57, 94, 98]. Genes associated with autism, such as UBE3B and ZNF18, are transcriptionally regulated by membrane depolarization and are involved in experience-dependent learning and synaptic plasticity [34]. These processes are also regulated by reelin in the cortex, and especially layer 1, throughout the lifespan in primates [72, 83]. Importantly, changes in axons in children and adults in autism were not accompanied by alterations in the overall density of neurons nor in the density of labeled inhibitory interneuron subclasses in layer 1 of LPFC, suggesting that these changes are specifically isolated to axon networks.

Previous examination of LPFC has revealed moderate changes in the expression of reelin in layer 1 of some individuals with autism [115]. Reelin, which is distributed throughout the soma and dendrites of most neurons in layer 1 [83], is secreted by Cajal-Retzius cells during prenatal development. Reelin has also been implicated in the regulation of synaptic plasticity and learning in the postnatal brain and is involved in the pathogenesis of neuropsychiatric disorders (reviewed in [37–39, 72]). Importantly, a study of the human temporal lobe found no change in the density of reelin-expressing neurons in autism: consistent with the findings reported here, this study further identified no change in neuron density in layer 1 in autism [14]. Taken together, our findings and previous reports suggest that reduction in the expression of reelin may be due to changes in protein processing or intracellular distribution within the cell population of layer 1 that can contribute to cortical patterning defects and postnatal changes in network structure in autism.

Other protein factors with altered expression in LPFC layer 1 in autism include chemokine ligand 14 (CXCL14) and neuron-derived neurotrophic factor (NDNF). CXCL14, an inflammatory cytokine, is involved in the regulation of myelination; reduction in the expression of this cytokine results in a reduction in myelination [4, 119], which is consistent with our findings of reduced myelination and increased branching in prefrontal cortices of adults with autism [129, 131, 133]. NDNF is mainly expressed by Cajal-Retzius cells of layer 1, and promotes the growth and development of neuronal cell bodies and neurite outgrowth [70, 106]. Disruption of any of these factors may adversely impact the development of the cortical column in LPFC, and could specifically underlie the atypical organization of circuits within layer 1 of LPFC in autism.

Placing our findings within the context of previous studies that have reported neuropathological and molecular changes in prefrontal cortices in autism offers additional clues about mechanisms that may underlie specific disruption of LPFC networks. Major feedback pathways that target layer 1 of LPFC and may contribute to the observed axon disorganization include pathways from high-order thalamic nuclei [60, 128] and, to a lesser extent, the amygdala [48], regions that are consistently affected in autism [8, 9, 51, 76, 107–109, 117, 118, 120]. Based on extensive experiments on a large cohort of subjects we have proposed that disruption of networks in autism depends on the time of the insult during prenatal development of cortical pathways [129]. A general, consistent theme that emerges from our findings is that feedback, short-range pathways from the deep layers of limbic cortices that target superficial layers of eulaminate cortices, which develop relatively early but mature late postnatally, are susceptible to disruption and are affected in autism [45, 129–131, 133]. We previously reported that in the neighboring ACC there is an increase in the expression of GAP-43 [129], a growth axon protein that is antagonistic to myelin basic protein [64] and promotes branching and shedding of myelin of axons. The ACC in primates is a major contributor of robust feedback pathways that terminate in the superficial layers of LPFC, including layer 1 [18, 20, 69, 85, 91, 131]. It is therefore conceivable that the observed axon pathology in layer 1 of LPFC may be due to disruption in this short-range feedback network linking ACC and LPFC [45]. This hypothesis is also supported by the oft reported over-connectivity of local frontal networks in autism [24, 113].

In conclusion, we systematically examined layer 1 of LPFC in individuals with and without autism at high resolution. We described the typical postnatal development and organization of axon circuits and local interneurons. Study of excitatory and inhibitory circuit components in parallel provided a novel framework that facilitated identification of pathological changes within cortical networks in autism. We found significant changes in the structure and organization of myelinated axons in LPFC layer 1 in individuals with autism, with important implications for the balance of excitation-inhibition and local cortical information processing. Our findings highlight feedback pathways in LPFC as an especially vulnerable node that underlies autism pathophysiology. Finally, our synthesis of the new findings with previous studies provide important clues that can help link the atypical development of frontal networks in autism with key molecular mechanisms and factors, whose interactions during development will need to be elucidated in future studies.
